# Magnetic Nanoparticles Create Hot Spots in Polymer Matrix for Controlled Drug Release

**DOI:** 10.3390/nano8100850

**Published:** 2018-10-18

**Authors:** Esther Cazares-Cortes, Maria Nerantzaki, Jérôme Fresnais, Claire Wilhelm, Nébéwia Griffete, Christine Ménager

**Affiliations:** 1 Sorbonne Université, CNRS, PHysico-Chimie des Electrolytes et Nanosystèmes InterfaciauX, PHENIX, F-75005 Paris, France; esther.cazares-cortes@espci.fr (E.C.-C.); maria.nerantzaki@sorbonne-universite.fr (M.N.); jerome.fresnais@sorbonne-universite.fr (J.F.); 2 Laboratoire Matière et Systèmes Complexes (MSC), UMR 7057, CNRS and Université Paris Diderot, 75205 Paris CEDEX 05, France; claire.wilhelm@univ-paris-diderot.fr

**Keywords:** magnetic nanoparticles, alternative magnetic field, thermosensitive polymer, molecularly imprinted polymer, drug release

## Abstract

Herein, original magnetic drug delivery nanomaterials for cancer therapy are developed and compared, with the purpose to show active control over drug release by using an alternative magnetic field (AMF). The rationale is to combine polymers and superparamagnetic nanoparticles to trigger such drug release under AMF. Two magnetic nanosystems are thus presented: magnetic nanogels made of thermosensitive and biocompatible polymers and core-shell nanoparticles with a magnetic core and a molecularly imprinted polymer as shell. Both encapsulate doxorubicin (DOX) and the DOX controlled release was investigated in vitro and in cells under AMF excitation. It confirms that the local heat profile at the vicinity of the iron oxide core can be used for the DOX controlled release. It also shows that both nanosystems help delivering more DOX inside the cells compared to internalization of free DOX. Finally, the DOX intracellular release could be remotely triggered under AMF, in athermal conditions, thus enhancing DOX cytotoxicity.

## 1. Introduction

Polymer-based nanoparticles have been largely studied as drug delivery systems (DDS) for cancer therapy [1], because polymers offer a large variety of chemical and physical properties for targeting [2], imaging [3] or drug release [4]. A smart combination of this versatile polymer matrix with inorganic nanoparticles leads to hybrid materials that can be stimulated on demand for remotely controlled drug delivery (RC-DDS). This can be useful in nanomedicine, as the payload can be released on demand with an enhancement of the therapeutic effectiveness and a reduction of the systemic toxicity. To achieve this goal, hybrid nanosystems have been designed, that contain an organic component such as a stimulus-responsive and/or biodegradable polymer and an inorganic component such as gold [5], maghemite [6], and silica nanoparticles [7] (NPs) to form RC-DDS. 

Iron oxide based magnetic nanoparticles (MNP) are extensively investigated in nanomedicine for their biocompatibility, their contrast agent properties [8] and their ability to generate heat when submitted to an alternative magnetic field (AMF) [9,10]. For the cancer field, their ability to generate heat when exposed to AMF of appropriate amplitude and frequency, opens interesting ways of treatment, using the MNP released heat to reduce the viability of cancer cells through apoptotic or necrotic pathways [11]. Such thermotherapy is already in clinical trials (e.g., phase II for glioblastoma multiforme, MagForce, Berlin, http://www.magforce.de/en/home.html) [12] Magnetic hyperthermia presents several advantages for cancer treatment like non-invasiveness, no depth penetration limit, remote controllability, nanoscale spatial resolution, and molecular-level specificity. However, this physical treatment still suffers from critical drawbacks, including a drastic reduction of the heat stimulation response when MNP are aggregated, the need for a high dose of MNPs, or therapeutic resistance [13].

A novel strategy concerning the use of magnetic hyperthermia is to take advantage of the heat generated at the vicinity of the MNP core without global heat dissipation. In this case, MNP act as individual “hot spot” and generate a localized thermal profile. Indirect proofs of this local heating have been made by several groups using different systems [14,15,16,17,18,19,20,21].

**Figure 1:**
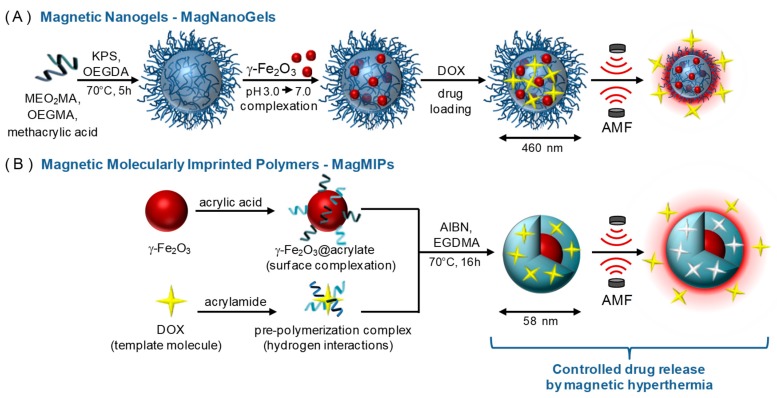
Schematic illustration of the synthesis of (**A**) MagNanoGels by precipitation radical copolymerization and post-assembly of MNPs inside nanogels and (**B**) MagMIPs via a subsequent grafting of an acrylic acid compound in the surface of MNPs and the growth of the polymer in the presence of DOX for imprinting polymerization. Loading and release of DOX under an AMF.

Here, we propose to take advantage of the local temperature increase under AMF to release a cancer drug (doxorubicin) using either a biocompatible thermosensitive nanogel that will release the drug through the conformation changes of the polymer network (Figure 1A) or a molecularly imprinted polymer that will release the drug by disruption of the hydrogen bonds existing between the drug and the polymer cage (Figure 1B). We aim at taking advantage of the hot spot effect under AMF of magnetic nanoparticles for controlled drug release. A comparison between both systems is proposed, to highlight the advantages and drawbacks of each.

## 2. Materials and Methods

### 2.1. Materials

Acetonitrile (ACN) (99.8%), ethanol (99.8%), hydrochloric acid (HCl) (37%), nitric acid (HNO_3_) (52%), iron (III) nitrate nonahydrate (Fe(NO_3_)_3_·9H_2_O, 99%), iron(III) chloride hexahydrate (FeCl_3_·6H_2_O,97%), acetone (Ac), diethyl ether (Et_2_O), acrylic acid (AA), ethylene glycol dimethylacrylate (EGDMA), and acrylamide (AAM), were supplied from Sigma-Aldrich Chemical Co. (Steinheim, Germany). Iron (II) chloride tetrahydrate (FeCl_2_·4H_2_O, 98%) Azo bis isobutyronirile (AIBN), ammonia (NH_4_OH) (20%) were purchased from Acros Organics (Halluin, France) for the synthesis of γ-Fe_2_O_3_ NPs and magnetic molecularly imprinted polymer (MagMIPs). Oligo-(ethylene glycol) methyl ether methacrylate (OEGMA; Mn = 500 g·mol^−1^), bis(ethylene glycol) methyl ether methacrylate (MEO_2_MA; Mn = 188.22 g·mol^−1^), oligo(ethylene glycol) diacrylate (OEGDA; Mn = 250 g·mol^−1^), methacrylic acid (MAA), potassium persulfate (KPS),potassium hydroxide (KOH; flakes), and nitric acid (HNO_3_; 52.5%) were purchased from (Sigma-Aldrich Chemical Co. (Steinheim, Germany) for the synthesis of bare and magnetic nanogels (MagNanoGels). HEPES hemisodium salt (dry powder) and doxorubicin hydrochloride (DOX) were purchased from Sigma-Aldrich Chemical Co. (Steinheim, Germany) and used for in vitro and intracellular drug release studies. Fluorescein, rhodamine B, 2-(dodecylthiocarbonothioylthio)-2-methylpropionic acid (TTMA) for preparing fluorescent MagMIPs and acryloxyethyl thiocarbamoyl rhodamine B for preparing fluorescent MagNanoGels, were purchased from Sigma-Aldrich Chemical Co. (Steinheim, Germany). Human prostate cancer PC-3 cells (CRL-1435) were supplied from American Type Culture Collection (ATCC) (Manassas USA). Dulbecco’s modified Eagle’s medium (DMEM) was purchased from Sigma-Aldrich Chemical Co. (Steinheim, Germany), while fetal bovine serum (FBS) penicillin and the Alamar Blue assay were purchased from Thermo Ficher Scientific (Carlsbad, CA, USA). All materials were used as received without any purification. Water was distilled and deionized.

### 2.2. Preparation of the Nanomaterials

#### 2.2.1. Synthesis of γ-Fe_2_O_3_ Nanoparticles

γ-Fe_2_O_3_ nanoparticles (MNP) were synthesized by coprecipitation of Fe^3+^ and Fe^2+^ ions according to Massart’s procedure [22]. Magnetite (Fe_3_O_4_) nanocrystals were prepared by coprecipitation of FeCl_3_ (1.6 mol) and FeCl_2_.4H_2_O (0.9 mol) salts in alkaline solution (NH_4_OH, 7 mol). The solid phase was separated from the supernatant by magnetic separation and immersed in a boiling solution (100 °C) of ferric nitrate (Fe(NO_3_)_3_, 0.8 mol) during 30 min to completely oxidize magnetite into maghemite (γ-Fe_2_O_3_). After magnetic decantation, 2 L of distilled water and 360 mL of HNO_3_ at 20%, were added to the solution and the mixture was stirred for 10 min. After several washing steps in acetone and diethylether to remove the excess of ions were performed. Prepared maghemite nanoparticles were suspended in water (1 L) at pH 1.5. To obtain the largest NPs, a size-sorting process was achieved by adding 20 mL of HNO_3_ (68%) to the ferrofluid and the mixture was stirred during 10 min [23]. A dense phase containing the biggest particles was separated from the supernatant containing the smallest ones. The dense phase was washed twice with acetone (200 mL) and twice with diethyl ether (200 mL). Then 100 mL of water was added to obtain the final dispersion. This fraction containing the largest nanoparticles was used to prepare MagMIPs and MagNanoGels. The volume fraction and average size of the maghemite MNP were determined by fitting the magnetization curve using Langevin’s law (Figure S1). The diameter of the particles was measured by TEM (d = 11.5 nm and σ = 0.33, Figure S2). The final iron content was checked by atomic absorption spectroscopy after degradation of the nanoparticles in acidic media (boiling HCl 35%).

#### 2.2.2. Preparation of MagNanoGels

Magnetic nanogels, denoted MagNanoGels, were prepared by loading preformed γ-Fe_2_O_3_ MNPs into thermoresponsive oligo(ethylene glycol) methyl ether methacrylate-based nanogels as previously described [24] and Figure 1. 

#### 2.2.3. Preparation of MagMIP Nanoparticles

We prepared γ-Fe_2_O_3_@DOX-MIP MNPs, labeled MagMIPs, according to Scheme 1-B as previously published [25] and Figure 1.

### 2.3. In Vitro Drug Release Experiments

In vitro DOX release of MagNanoGels and MagMIPs was analyzed in different conditions to assess the influence of the temperature and the AMF. We monitored DOX release in medium at pH 7.5 (0.1 M sodium HEPES solution). For all experiments, DOX−MagNanoGel solution (1 mL, [Fe] = 8.4 mM, [nanogel] = 2.94 mg·mL^−1^, [DOX] = 69.5 μM, loading efficiency 63%) and MagMIPs (2 mL, [Fe] = 50 mM, [DOX] = 13 µM, [MagMIPs] = 0.5 mg mL^−1^) were placed in two Eppendorf tubes and heated at 37 °C (human body temperature) during 4 h. Each 30 min, the supernatant was collected by magnetic separation (5 min) and the amount of DOX released was quantified by UV−VIS spectroscopy. We remark that if we use the same quantity of materials (MagNanogels or MagMIPs), the DOX concentration that is encapsulated in the polymer is in the same order of magnitude. 

### 2.4. Cellular Drug Release Experiments

Human Prostatic Cancer Cells (PC-3, ATCC^®^ CRL-1435™) were cultured at 37 °C in 5% CO^2^ in Dulbecco’s modified Eagle’s medium (DMEM) completed with 10% fetal bovine serum and 1% penicillin and streptomycin antibiotics. MagMIP and MagNanoGels were incubated with the cells for 2 h at iron concentrations in the range [Fe] = 0.5–2 mM. Control Fe2O3@citrate NPs (same iron oxide core with simple citrate coating) were incubated for the same 2 h time period, at [Fe] = 2 mM. Incubation was followed by an overnight chasing period in complete medium. For confocal microscopy observation, cells membranes were then labeled by PKH26 red dye, cells were fixed right after with 2% paraformaldehyde, and in some cases poststained with DAPI (4′,6-diamidino-2-phenylindole) to localize the nucleus. For AMF-induced doxorubicin release in the cellular environment, cells were exposed to the alternative magnetic field (regulated at 37 °C) for 30 min, 1 h 30 min, and 2 h 30 min. The AMF treatment recorded no macroscopic heating. Viability measurements were performed the day after using Alamar Blue colorimetric assay (Life Technologies).

### 2.5. Instrumentation

γ-Fe_2_O_3_ MNPs, MagMIPs, and MagNanoGels were observed using a JEOL-100 CX TEM (Croissy-sur- Seine, France). A droplet of diluted nanoparticles suspension in water was deposited on a carbon coated copper grid and the excess was drained using filter paper. Size analysis was achieved on TEM images using ImageJ software (https://imagej.nih.gov/ij/)

Hydrodynamic diameter (d_h_) and zeta potential measurements were recorded using a Malvern Instruments Nanosizer (Malvern, UK). The magnetic properties of γ-Fe_2_O_3_ MNP were measured with a Vibrating Sample Magnetometer. Using a diluted, size-sorted aqueous solution of γ-Fe_2_O_3_ MNP (volume fraction of MNP ϕ < 2%); and assuming a lognormal distribution P(d), the magnetic diameter (d_0_) and the polydispersity index (σ) of MNPs solution were deduced by fitting the magnetization curve with Langevin’s function model. Fourier transform infrared (FT-IR.) spectra of γ-Fe_2_O_3_ MNPs and Fe_2_O_3_@DOX-MIP MNPs were recorded on a Bruker Tensor 27 spectrometer (Bruker, France) on pressed KBr pellets. Spectra were obtained at regular time intervals in the MIR Region of 4000–400 cm^−1^ at a resolution of 4 cm^−1^ and analyzed using OPUS software. 

Hyperthermia experiments for nanoparticles in suspension were conducted on a magneTherm apparatus (magneTherm AC system, Nanotherics Corp., Newcastle under Lyme, UK) at 335 kHz and 9 mT. The sample was thermalized at 37 °C before the application of the alternative magnetic field. The temperature was probed using a fluorooptic fiber thermometer. 

For the cell study, we used a home-made magnetothermal setup consisting of a resonant RLC circuit connected to a copper coil in which the temperature was kept constant at 37 °C for the MagMIPs and a Nanoscale Biomagnetics for the MagNanoGels nanoparticles 

Absorbance measurements were achieved with an Avantes UV–VIS spectrophotometer, with 100 μm in diameter optical fibers. UV–VIS measurements were configured with a range from 200 to 1100 nm. A combined deuterium-halogen light source was used. 

## 3. Results and Discussion

### 3.1. Characterization of the Magnetic Nanomaterials

#### 3.1.1. Characterization of γ-Fe_2_O_3_ MNPs

The magnetization curve of MNPs confirms their superparamagnetic properties (Figure S1). TEM analysis shows magnetic nanoparticles with an average particle diameter (d_0_) of 11.5 nm and a polydispersity (σ) of 0.33 (Figure S2). The heating efficiency of the iron oxide nanoparticles core ([Fe] = 50 mM, 300 s AMF application at 25 °C) was measured at 342 kHz and different magnetic field intensities (4.8, 9, 13.5, and 18 mT) (Table 1). The heating power is expressed in terms of specific absorption rate (SAR). It is obtained from the initial slope of temperature curves and it varies with the external magnetic field parameters. SAR increases with the amplitude and frequency of the magnetic field.

#### 3.1.2. Characterization of the MagMIP Nanoparticles

**Figure 2:**
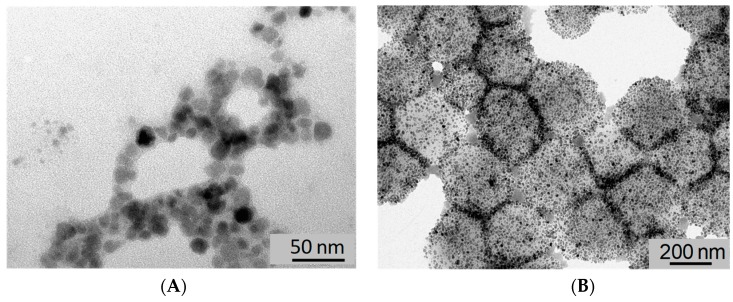
TEM images of (**A**) MagMIPs and (**B**) MagNanoGels loaded with 37.5 wt% of γ-Fe_2_O_3_.

The polyacrylamide shell of the MagMIPs is difficult to visualize on TEM images (Figure 2A) due to the low contrast of the polymer and the weak thickness of the shell however the thin layer is evidenced by IR spectroscopy (Figure S3a) with the presence of new peaks compared to particles before polymerization. These peaks are detected at 3455 (NH_2_), 2904 (CH_2_), and 1746 cm^−1^ (C=O). The presence of the polymer shell is also evidenced by the increase of the hydrodynamic diameter after polymerization, as illustrated by dynamic light scattering (DLS) measurements, revealing an increase from 27 to 58 nm (PDI = 0.28 and 0.66) for γ-Fe_2_O_3_ and MagMIPs, respectively (Figure S3b). The zeta potential of MagMIPs is −27 mV whatever the pH. Finally, thermogravimetric analysis (TGA) shows that the amount of polyacrylamide (PAAM) on MagMIPs was about 70% of the total particle weight, as determined from the significant mass change between 420 and 470 °C owing to decomposition of PAAM (Figure S4b).

#### 3.1.3. Characterization of the MagNanogels

The assembly of preformed γ-Fe_2_O_3_ MNP inside nanogels provides monodisperse magnetic nanogels with a high content of MNPs as image by TEM (d = 584 nm and PDI = 0.371, Figure 2B). If the mass ratio is equal or less than 37.5 wt%, MagNanoGels are stable in distilled water. The MNPs are adsorbed inside nanogels by complexation of carboxylic acid groups of the polymer matrix onto the surface of MNP. Nanogels are negatively charged in distilled water (ζ = −35.4 mV) due to the presence of carboxylate groups. The TGA curve of bare nanogels shows a significant decrease from 225 to 430 °C, due to the thermal decomposition of the polymer matrix; the total weight loss is reached above 450 °C. Furthermore, the TGA curve of MagNanoGels confirms the presence of MNP in the nanogels. Above 450 °C, the maximum weight loss reaches 64% owing to decomposition of polymers chains; therefore, 36% of MNPs were incorporated into nanogels (Figure S4a). These MagNanoGels are thermoresponsive due to MEO_2_MA and OEGMA monomers as previously studied in [24].

### 3.2. DOX Release Triggered by AMF

For magnetic hyperthermia drug release experiments, DOX-MagNanoGel solution (1 mL, [Fe] = 8.4 mM, [nanogel] = 2.94 mg mL^−1^, [DOX] = 69.5 μM) and DOX-MAG-MIP (2 mL, [Fe] = 50 mM, [DOX] = 13 µmol L^−1^) were placed in two Eppendorf tubes inside the coil of a MagneTherm system, and an AMF was applied by pulses of 30 min (335 kHz, 9 mT, 12.0 kA·m^−1^). After each AMF pulse, the supernatant was collected by magnetic separation (5 min). The amount of DOX released in each supernatant was quantified by UV−VIS spectroscopy.

**Figure 3:**
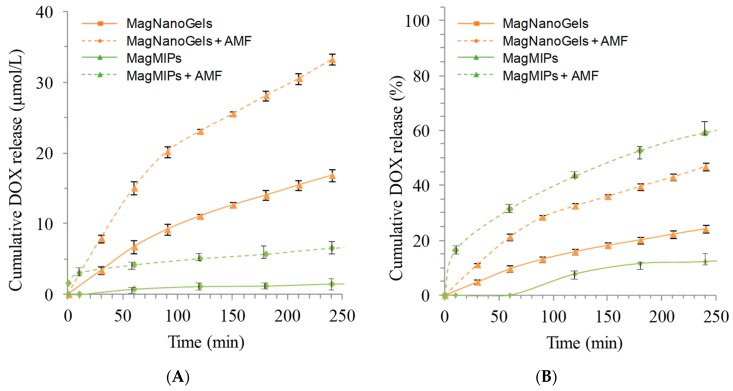
Cumulative DOX release at pH 7.5 (**A**) in μmol L^−1^ and (**B**) in percent versus time of MagMIP and MagNanogels at 37 °C without magnetic field (full lines) and under AMF (342 kHz, 9 mT, dotted lines).

The MagMIPs exposed to the AMF released 7 μM (60%) of DOX after 4 h at 37 °C whereas a sample left for 4 h at 37 °C only shows reduced DOX release (1 µM, 10%) (Figure 3A). More interestingly, for each time point, the drug release is significantly higher when the MagMIPs are subjected to AMF. For the MagNanoGels, after 4 h, at 37 °C and pH 7.5, 16.7 μM (24%) of DOX was released, whereas, under AMF (335 kHz, 9 mT, 12.0 kA m^−1^, 30 min), DOX release was significantly enhanced (32.7 μM, 45%) (Figure 3A). MagNanoGels release two-fold more drug under the AMF. If we compare these two materials, MagMIPs do not release a high quantity of DOX compared to MagNanoGels (7 µM and 16.7 µM) but in terms of percent the MagMIPs release more than half of the DOX contained inside the matrix (Figure 3B). Additionaly, even if MagNanoGels release a very large quantity of DOX compared to the MagMIPs, this later has a low passive release (only 10% of DOX is release without AMF at 37 °C and pH = 7.5 compared to 24% for MagNanoGels in similar conditions). In the case of MagMIPs, the DOX interacts with the polymer matrix by forming hydrogen bonds, whereas in the case of MagNanoGels the drug is only physically entrapped (Figure 3B).

These two systems can answer different applications. For instance, MagMIPs can reduce significantly the non-specific release and deliver on-demand the DOX, while MagNanoGels could work as giant reservoirs that deliver continuously the DOX, with additional delivering peaks under local activation through AMF.

To evaluate the local temperature generated by MNP, we compared the DOX release under AMF to experiments achieved at different macroscopic temperatures.

### 3.3. DOX Release Triggered by Temperature 

**Figure 4:**
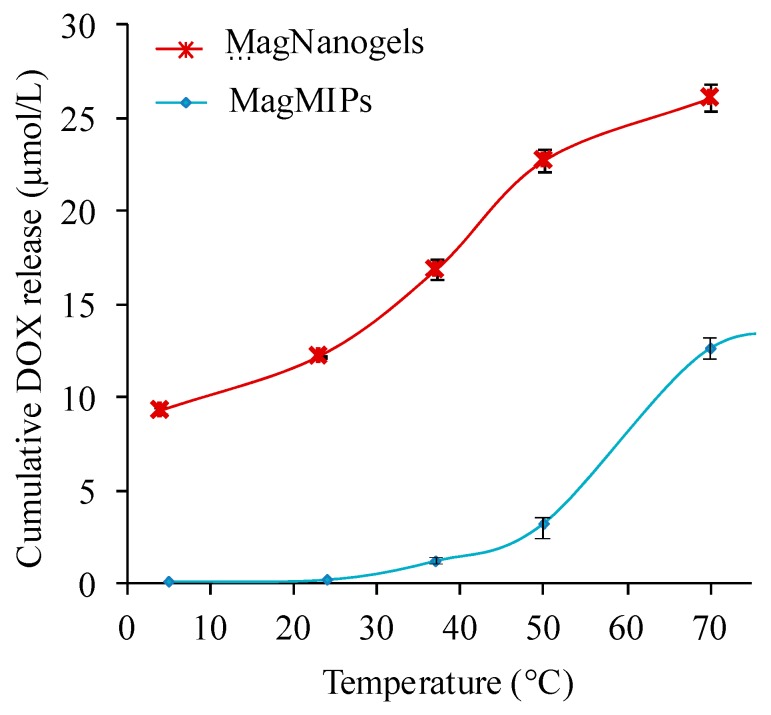
Influence of the temperature (water bath) on the amount of DOX released from MagMIPs ([Fe] = 50 mM) and MagNanoGels ([Fe] = 8.4 mM). Cumulative DOX release (µmol/L) after 4 h at the desired temperature.

To evaluate the temperature effect on the drug-release profile, MagMIPs and MagNanoGels were heated at different temperatures (from 4 to 70 °C) during 4 h and the supernatants were analyzed after magnetic separation. To reach the desired temperature, the samples were placed in a thermalized water bath. Each release experiment was carried out in triplicate, and average values were calculated and used to plot cumulative DOX release profiles. As shown in Figure 4, the cumulative DOX release increases when the global temperature rises. After 4 h, MagMIPs release 0.9, 1.5, 2.8, and 12.2 µM and MagNanoGels release 11.8, 16.7, 22.2, and 25.0 μM of DOX at, respectively, 23, 37, 50, and 70 °C. As the macroscopic temperature elevation measured during AMF experiments is negligible, therefore, we can conclude that the effect of the AMF is due to high temperature profiles from the nanoparticle surface. This profile is restricted to the close vicinity of the MNP, thus leading to localized heating. MagMIPs demonstrate a core-shell structure while MagNanoGels is more likely a gel in which hot-spots are distributed. They both benefit from local heating effects.

When applying AMF, the global temperature of MagNanoGels and MagMIPs solutions remains as initially set at 37 °C. The curves of Figure 4 were used as calibration curves to acquire a quantitative correlation between DOX release and the local temperature under the AMF. An interesting point is that for both systems the local temperature is estimated around 65–70 °C. It is important to note that we compare two systems with different iron concentrations. However, as MNP are used as a local heater the drug release must not depend on the iron concentration. The drug release occurs when the system reaches the volume phase transition of the thermosensitive nanogels (VPTT 47 °C at pH 7.7) or when the energy supplied is able to break the hydrogen bonds existing between the drug and the polymer matrix in the case of MagMIPs.

### 3.4. Intracellular Drug Release Experiments

**Figure 5:**
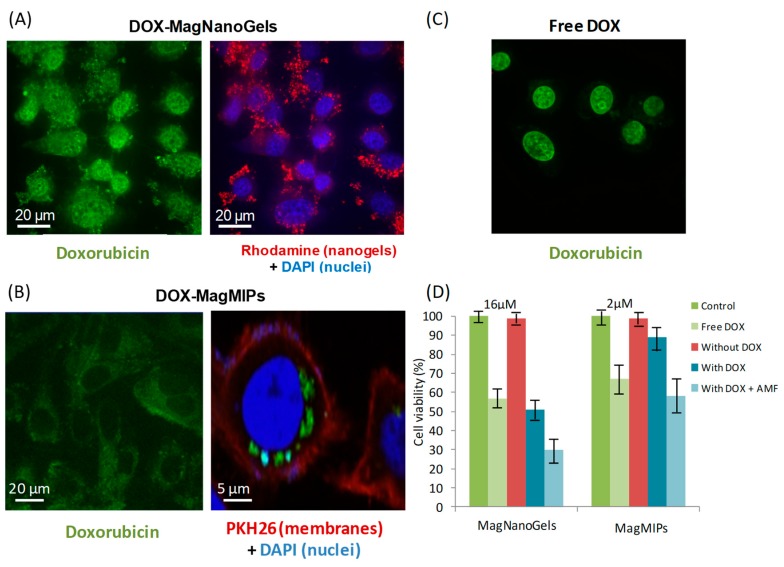
Confocal imaging of tumor cells (PC-3) having internalized (**A**) MagNanoGels-37.5 wt% (2 h incubation at [Fe] = 2 mM and [DOX] = 16 µM) and (**B**) DOX-MagMIPs nanoparticles (2 h incubation at [Fe] = 2 mM and [DOX] = 1.3 µM. DOX is detected in the green channel (excitation at 488 nm, emission at 561 nm). Nuclei are stained by DAPI in blue (excitation at 405 nm). For MagMIPs studies cell membranes are stained by PKH26 in red; while for MagNanoGels studies, only nanogels are in red by covalent bonding to rhodamine (excitation at 561 nm). For comparison, (**C**) cells incubated for 2 h with free DOX (16 µM). Z reconstruction identify DOX inside the cells. (D) Cell viabilities for free DOX at [DOX] = 2 µM and 16 µM, for DOX-MagNanoGels at [Fe] = 2 mM and [DOX] = 16 µM and for DOX-MagMIPs at [Fe] = 2 mM and [DOX] = 1.3 µM.

DOX release was tested in cancer cells, foreseeing future therapeutic application. DOX-MagNanoGels and DOX- MagMIPs were very efficiently captured by cancer cells (PC-3), with an iron load per cell of respectively 15 pg and 10 pg, when incubation (2 h) was performed at [Fe] = 2 mM. The internalization did not induce any toxic cellular response, demonstrating the correct biocompatibility of those materials without DOX. For MagNanoGels and MagMIPs encapsulating DOX, DOX was clearly associated with the tumor cells on confocal microscopy images, demonstrating an intracellular localization, sequestered inside intracellular endosome-like compartments (Figure 5A–C). Cell viability was more impacted by the internalization of the MagNanoGels containing DOX than MagMIPs associated with DOX (54% versus 88% cell viability, respectively, Figure 5D). Note, however, that for the same iron concentration (2 mM), the DOX content was higher in MagNanoGels than in MagMIPs ([DOX] = 16 µM versus [DOX] = 1.3 µM). In both cases, the cell viability was significantly lowered after AMF stimulation (2 h). It was reduced from 54% to 30% when applying AMF for DOX-MagNanoGels (with [DOX] = 16 µM) and from 88% to 60% for DOX-MagMIPs (but with lower [DOX] = 1.3 µM) (Figure 5D). These results confirm that the payload is continuously released for MagNanoGels, but can be delivered in larger amounts under AMF. By contrast, when DOX is bonded to the MIP, DOX is inactive. Interestingly, it also demonstrates that both nanomaterials favor the internalization of DOX, as the final therapeutic effect is much more pronounced under AMF that for the equivalent dose of free DOX internalized in cells. For DOX-MagNanoGels, viability under AMF is 32% while higher at 58% for free-DOX at the same incubation concentration; and for DOX-MagMIPs, the viability under AMF reached 59%, while still at 92% for free-DOX at same dose. These results are in agreement with intracellular drug release experiments showing a lower passive diffusion for DOX-MagMIPs and a higher DOX concentration release for DOX-MagNanoGels, still under athermal conditions. Indeed, during the AMF exposure, the cell medium was maintained at 37 °C. Altogether, these cellular experiments support the DOX release under AMF application evidenced in solution and demonstrate the possibility of initiating a chemotherapeutic treatment via an athermal magnetic hyperthermia strategy.

## 4. Conclusions

To conclude, we demonstrated that, for two different hybrid magnetic materials, it is possible to tune the release of a drug in athermal conditions thanks to local heating. For MagMIPs, the passive release is very low, but the amount of DOX encapsulation and subsequent release is limited. For MagNanoGels, unspecific release is larger compared to the previous systems, but it can deliver large quantities of DOX. For in vitro doxorubicin release, even if the macroscopic temperature was maintained at 37 °C during all the AMF experiments, we estimated that the temperature inside the polymer matrix was close to 60 °C. Cancer cells experiments showed the efficiency of DOX is increased under AMF due to local heating of the magnetic nanoparticles in hybrid systems. DOX is released from both magnetic materials under AMF, increasing their efficiency and reducing cell viability.

Altogether, these results show that it is possible to tune the release of a drug depending of the final application targeted. Indeed, selecting MagNanoGels, it is possible to release continuously a drug with adaptive increase of the release under AMF. Selecting MagMIPs reduces drastically the passive release and allows an on-demand release of smaller quantities under AMF. Thus, combining both systems into a single nanoplate form through a smart design could combine the advantages of both individual systems, for instance, by covering high payload nanogels with a more impermeable MIP layer.

## Figures and Tables

**Table 1:** Heating efficiency (SAR, in W per gram of iron) of γ-Fe_2_O_3_ MNP (2 mL, [Fe] = 0.05 mol L^−1^) under AMF at 342 kHz and different magnetic field intensities (4.8, 9, 13.5 and 18 mT).

Magnetic Field Amplitude (mT)	SAR (W/g Fe)
4.8	0
9	21
13.5	52
18	73

## Supplementary Materials

The following are available online at http://www.mdpi.com/2079-4991/8/10/850/s1, Figure S1. (a) Magnetization curve of—Fe_2_O_3_ NPs at 298 K measured by VSM. (b) Size distribution modeled from the experimental data of (a) with a lognormal law (Langevin’s function model); Figure S2. (a) TEM image of bare, size sorted γ-Fe_2_O_3_ NPs. (b) Size distribution of γ-Fe_2_O_3_ NPs obtained by TEM image (n = 200 NPs; log-normal distribution model (red line) with d_0_ = 11.5 nm and σ = 0.33; Figure S3. (a) FT-IR spectra and (b) size distribution from DLS of bare—Fe_2_O_3_ (a) and Mag-MIP nanoparticles (b); Figure S4. Thermogravimetric analysis (N_2_(g); 10 °C·min^−1^) of (a,b) bare—Fe_2_O_3_ magnetic nanoparticles; (a) MagNanoGels-X wt% loaded with X = 0 and 37.5 wt% MNPs; and (b) MagMIPs.
