# Supplementary material for: Magnetic Nanoparticles Create Hot Spots in Polymer Matrix for Controlled Drug Release
Source: Nanomaterials (Basel). 2018 Oct 18;8(10):850. doi: 10.3390/nano8100850 (PMC6215271; doi:10.3390/nano8100850)
Supplement: Supplementary file 1 [file nanomaterials-08-00850-s001.pdf]

Supporting Information

Magnetic Nanoparticles Create Hot Spots in Polymer Matrix for Controlled Drug Release

Esther Cazares-Cortes[1], Maria Nerantzaki[1], Jérôme Fresnais[1], Claire Wilhelm[2], Nebewia Griffete[1,*] and Christine Ménager[1,*]

[1] Sorbonne Université, CNRS, PHysico-chimie des Electrolytes et Nanosystèmes InterfaciauX, PHENIX, F-75005 Paris, France.

[2] Laboratoire Matière et Systèmes Complexes (MSC), UMR 7057, CNRS and Université Paris Diderot, 75205 Paris Cedex 05, France

* Correspondance : nebewia.griffete@sorbonne-universite.fr; christine.menager@sorbonne-universite.fr

Magnetization Measurement

The magnetization curve M(H) (Figure S1) of a suspension of monodisperse γ-Fe_2O_3 NPs can be described by Langevin's law. Thus, fitting the Langevin curve to the experimental magnetization curve and assuming a log-normal distribution P(d) (Equatoin (S1)), the magnetic diameter (d_0) and the polydispersity index (σ) of MNPs solutions are calculated:

$$P(d) = \frac{1}{\sqrt{2\times\pi}\times\sigma\times d} \times exp\left[-\frac{ln^2(d/d_0)}{2\times\sigma^2}\right] \quad \text{(S1)}$$

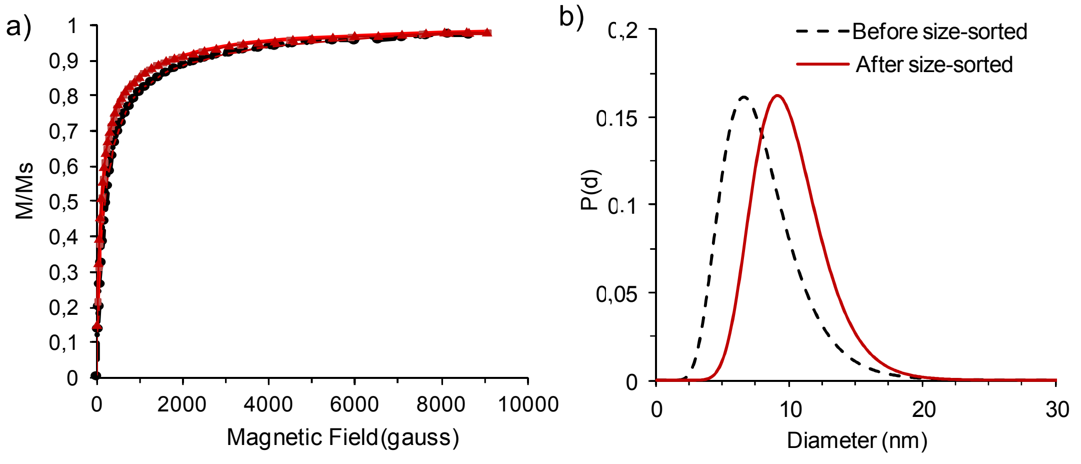

Figure S1. (**a**) Magnetization curve of γ-Fe_2O_3 NPs (red points, Langevin model; black points, experimental curve) at 298 K, measured by VSM. (**b**) Size distribution before and after size-sorting modeled from the experimental data of (**a**) with a lognormal law (Langevin's function model).

TEM Analysis

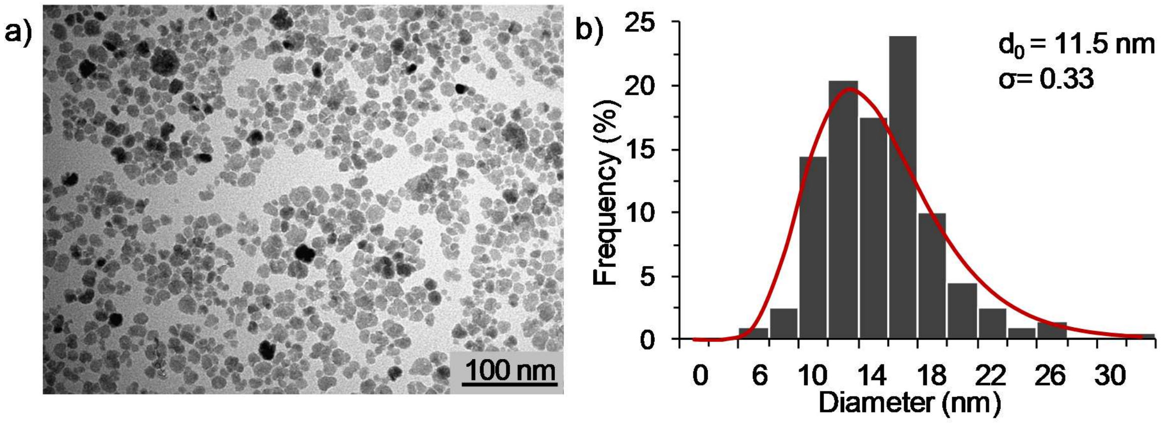

Figure S2. (**a**) TEM image of bare, size sorted γ-Fe_2O_3 NPs. (**b**) Size distribution of γ-Fe_2O_3 NPs obtained by TEM image analysis (n = 200 NPs; log-normal distribution model (red line) with d_0 = 11.5 nm and σ = 0.33.

FTIR and DLS on MagMIP Nanoparticles

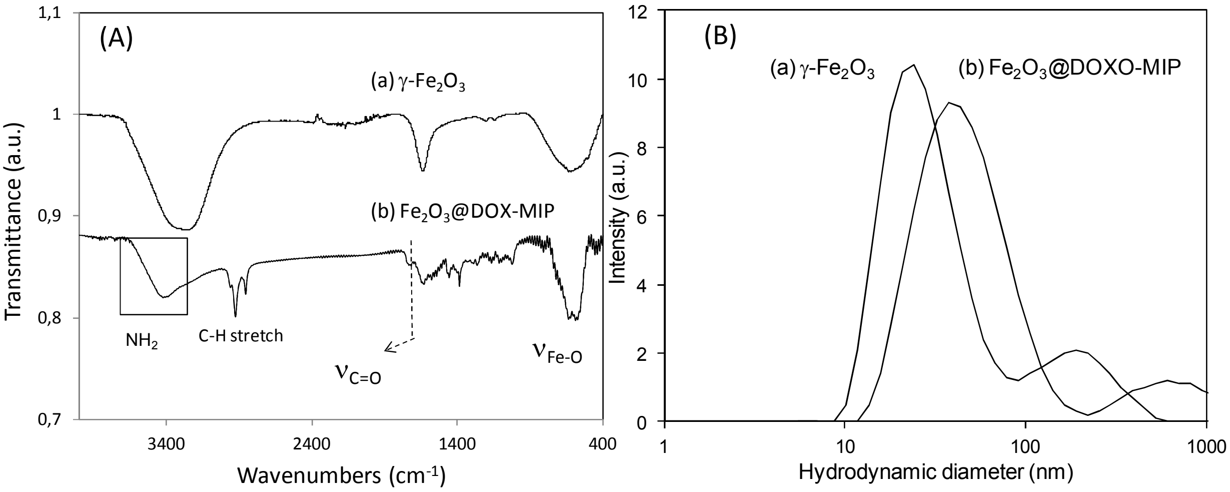

Figure S3. (**A**) FT-IR spectra and (**B**) size distribution from DLS of bare γ-Fe_2O_3 (a) and MagMIP nanoparticles (b).

ATG Curve of MagNanogels and MagMIP

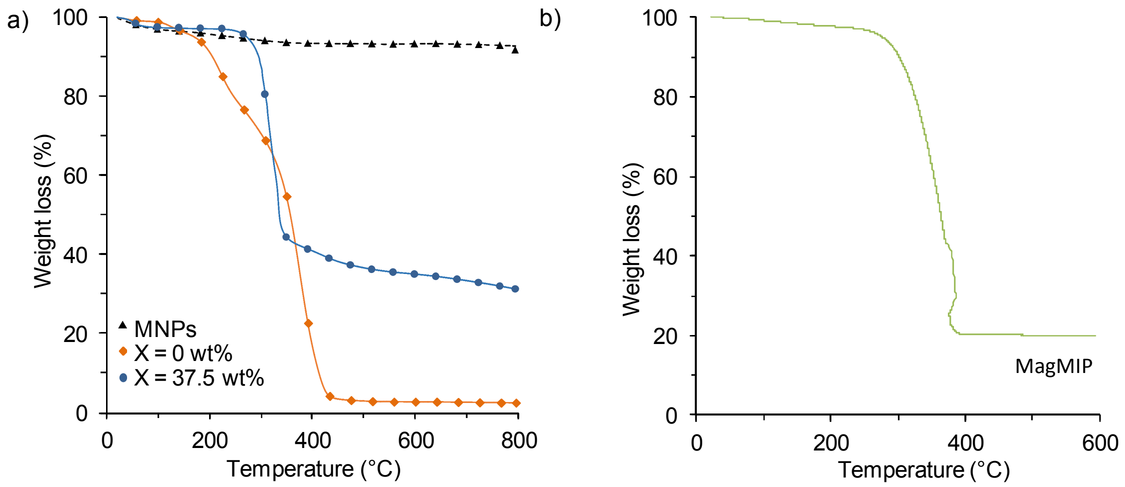

Figure S4. Thermogravimetric analysis ($N_{2(g)}$; 10 °C.min^{-1}) of (a,b) γ-Fe_2O_3 magnetic nanoparticles; (**a**) MagNanoGels-Xt% loaded with X = 0 and 37.5 wt% MNPs and (**b**) MagMIPs
